# Differential association of key bacterial groups with diatoms and *Phaeocystis spp*. during spring blooms in the Southern Ocean

**DOI:** 10.1002/mbo3.1428

**Published:** 2024-08-09

**Authors:** Nyree J. West, Marine Landa, Ingrid Obernosterer

**Affiliations:** ^1^ CNRS FR3724, Observatoire Océanologique de Banyuls (OOB) Sorbonne Université Banyuls sur mer France; ^2^ Laboratoire d'Océanographie Microbienne, LOMIC, CNRS Sorbonne Université Banyuls sur mer France

**Keywords:** algae, cell‐microbe interactions, molecular microbial ecology, Ocean microbiology

## Abstract

Interactions between phytoplankton and heterotrophic bacteria significantly influence the cycling of organic carbon in the ocean, with many of these interactions occurring at the micrometer scale. We explored potential associations between specific phytoplankton and bacteria in two size fractions, 0.8–3 µm and larger than 3 µm, at three naturally iron‐fertilized stations and one high nutrient low chlorophyll station in the Southern Ocean. The composition of phytoplankton and bacterial communities was determined by sequencing the *rbcL* gene and 16S rRNA gene from DNA and RNA extracts, which represent presence and potential activity, respectively. Diatoms, particularly *Thalassiosira*, contributed significantly to the DNA sequences in the larger size fractions, while haptophytes were dominant in the smaller size fraction. Correlation analysis between the most abundant phytoplankton and bacterial operational taxonomic units revealed strong correlations between *Phaeocystis* and picoeukaryotes with SAR11, SAR116, *Magnetospira*, and *Planktomarina*. In contrast, most *Thalassiosira* operational taxonomic units showed the highest correlations with *Polaribacter*, *Sulfitobacteria*, *Erythrobacter*, and *Sphingobium*, while *Fragilariopsis*, *Haslea*, and *Thalassionema* were correlated with OM60, *Fluviicola*, and *Ulvibacter*. Our in‐situ observations suggest distinct associations between phytoplankton and bacterial taxa, which could play crucial roles in nutrient cycling in the Southern Ocean.

## Graphical abstract

We used rbcL and 16S rRNA gene sequencing to characterize phytoplankton and bacterial communities in size‐fractionated samples. Our correlation analysis between the most abundant phytoplankton and bacterial operational taxonomic units revealed distinct bacterial groups associated with the dimethylsulfoniopropionate (DMSP) producer *Phaeocystis*, including SAR11, SAR116, *Magnetospira*, and *Planktomarina*, or the diatom *Thalassiosira*, including *Polaribacter*, *Sulfitobacteria*, *Erythrobacter*, and *Sphingobium*. These results suggest unique associations between phytoplankton and bacterial taxa, which could play significant roles in nutrient cycling in the Southern Ocean.

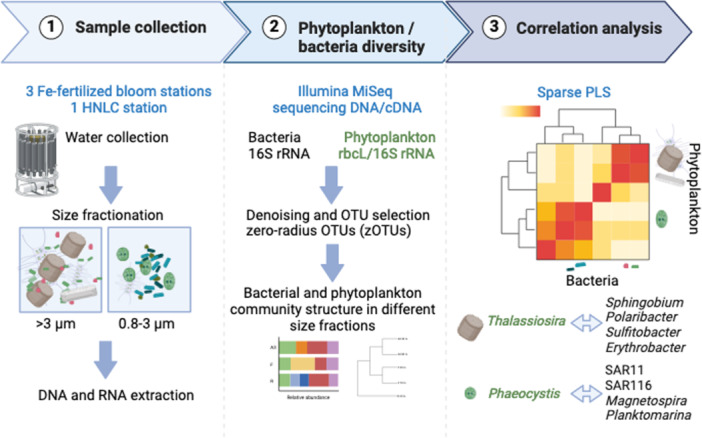

## 1 INTRODUCTION

The Southern Ocean (SO) plays a major role in global climate regulation through both physical and biological mechanisms, notably by the contribution of phytoplankton to the carbon cycle via the biological carbon pump and to the sulphur cycle through dimethylsulfoniopropionate (DMSP) production. Phytoplankton groups contribute differently to these processes, with diatoms being key players in the SO primary production and CO_2_ drawdown (Sarthou et al., 2005) while prymnesiophytes are globally important DMSP producers (Keller et al., 1989; McParland & Levine, 2019). Besides their specific roles in elemental cycles, these groups of phytoplankton could differently affect food web structures in the SO (Hunt et al., 2021; Krumhardt et al., 2022). Phytoplankton cell size in particular influences grazing, therefore playing a key role in the transfer of cell material toward higher trophic levels and the efficiency of the biological carbon pump (Christaki et al., 2021; Eddy et al., 2021; Moline et al., 2004). For these reasons, the composition and activity of phytoplankton communities are critical determinants of SO ecosystem functioning.

The SO is the largest high nutrient low chlorophyll (HNLC) region of the world's oceans with low phytoplankton biomass during most of the year due to limitation principally by the micronutrient iron (Fe). Spring phytoplankton blooms can, however, develop in regions with natural Fe input, such as over continental shelves or close to islands such as the Kerguelen, Crozet, and Sandwich Islands (Blain et al., 2007; Pollard et al., 2007) and are usually dominated by diatoms (Quéguiner, 2013). The prevalence of diatoms in these naturally Fe‐fertilized regions is largely driven by the availability of silicic acid and diatoms' ability to outcompete other phytoplankton taxa for available iron (Marchetti et al., 2012). The onset of silica limitation and subsequent decline of the diatom blooms are usually accompanied by a phytoplankton shift from small, fast‐growing to large, slow‐growing diatoms (Blain et al., 2021) to an increasing contribution of haptophytes dominated by *Phaeocystis* spp. (Irion et al., 2021; Salter et al., 2007). *Phaeocystis* is generally more abundant in coastal regions (Davidson et al., 2010) and can form extensive prolonged blooms as was reported north‐west of the Ross Sea above the Australian Antarctic Ridge (AAR) (Schine et al., 2021) and north of the Crozet plateau (Poulton et al., 2007). Shifts in environmental conditions and ensuing changes in phytoplankton assemblages are expected to impact other components of the microbial community with repercussions on biogeochemical cycles and food web structure.

There is overwhelming evidence of ecological coupling between phytoplankton and heterotrophic bacteria through studies carried out both in the field and the lab. Successional changes through the course of phytoplankton blooms are mirrored in the heterotrophic bacterial community composition (see reviews by Buchan et al., 2014; Bunse & Pinhassi, 2017) that can reveal repeated patterns on multi‐annual scales (Fuhrman et al., 2006; Lambert et al., 2019). These interactions may range from simple resource provision to highly specific associations encompassing a multitude of interactions from the mutualistic exchange of info‐chemicals or specific nutrients to competition and antagonism (Cirri & Pohnert, 2019; Costas‐Selas et al., 2024; Coyne et al., 2022). For some of these exchanges to occur, spatial proximity of the partners has long been hypothesized (Bell & Mitchell, 1972) which led to the coining of the term “phycosphere,” analogous to the rhizosphere in plant‐soil bacteria interactions (see (Seymour et al., 2017) for a review). In the phycosphere, high phytoplankton‐derived metabolite concentrations provide a dissolved organic matter (DOM) hotspot that facilitates bacterial growth, particularly for bacteria that are chemotactic or require attachment to their eukaryotic partners. The phycosphere depends on phytoplankton cell size and exudation rates, with large (>50 µm), leaky species expected to provide a much larger phycosphere than picocyanobacteria that are estimated to fall below the theoretical lower limit of detection by chemotactic bacteria (Seymour et al., 2017). Hence, distinct phytoplankton communities with specific phycosphere properties can exert different selective pressure on coexisting bacterial taxa.

The naturally fertilized region east of Kerguelen Island has been extensively used as a natural laboratory to investigate the effect of Fe on biogeochemistry and ecosystem functioning. Pronounced and specific responses of the bacterial community during the different phases of the Fe‐induced phytoplankton blooms were observed (Hernandez‐Magana et al., 2021; Landa et al., 2016; Obernosterer et al., 2011; West et al., 2008). However, these previous studies focused mostly on free‐living bacteria (0.2–0.8 µm). The major aim of the present study was to identify associations between phytoplankton and particle‐attached bacteria by focusing on two size fractions (0.8–3 and >3 µm) that should separate the larger and smaller size classes of phytoplankton (Biggs et al., 2019) and the results presented in this study complement observations in the free‐living fraction from the same period and study sites (Dinasquet et al., 2022). To achieve this objective, heterotrophic bacteria and phytoplankton diversity and activity were assessed in spring phytoplankton blooms by metabarcoding of the 16S rRNA gene for bacteria and the RubisCO *rbcL* gene for phytoplankton. As a proxy for activity (protein synthesizing potential; (Blazewicz et al., 2013), cDNA fragments generated from RNA from each marker gene were also analyzed by the same approach.

## 2 EXPERIMENTAL PROCEDURES

### 2.1 Study area

**Figure 1:**
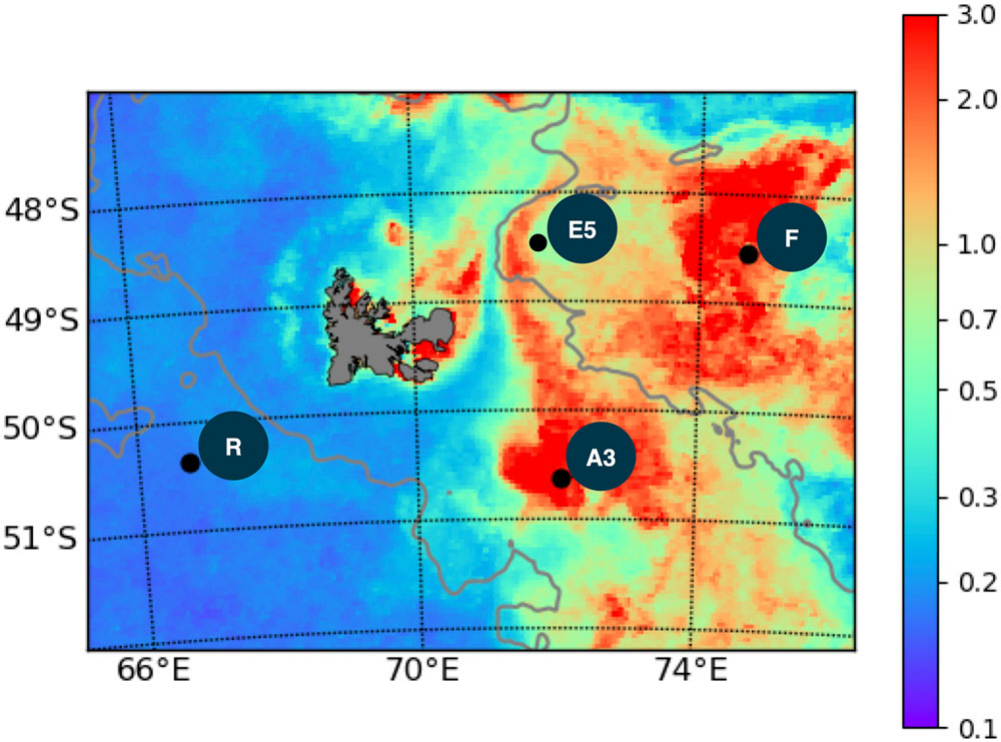
Location of the four sampling stations with MODIS‐Aqua satellite (CLS‐CNES) images of surface chlorophyll a (chla) concentrations (µg/L) indicating the presence of the phytoplankton blooms. A3 is the reference bloom station above the Kerguelen Plateau and R is the reference HNLC station. Station F was situated in a bloom above the Polar Front and E5 was located in the Polar Front meander.

The samples for the present study were collected in the naturally Fe‐fertilized and high nutrient low chlorophyll (HNLC) waters off Kerguelen Island during the KEOPS2 (Kerguelen Ocean and Plateau Study 2) cruise aboard the French R/V Marion Dufresne II ship in the austral spring (10 October to 20 November 2011) (Figure 1).

### 2.2 Sample collection

Seawater samples were collected from the four stations at 3–4 discrete depths using a CTD equipped with 12L Niskin bottles (General Oceanics) (See Table 1). One set of samples from the upper 3 depths (20–150/160 m) were size fractionated by prefiltering 9L of seawater through a 65 µm mesh and then filtering onto 10 µm pore‐size polycarbonate filters (PC, Nuclepore, 47 mm diameter). The second set of samples was collected from 4 depths (20–300 m), and passed through a 25 µm mesh, before sequentially filtering onto 3 µm and 0.8 µm PC filters followed by a 0.2 µm Sterivex cartridge filter. The volumes filtered ranged between 5 and 7 L except for the 20 and 300 m depths at station R where 2.5 and 3 L were filtered respectively. All filters were stored frozen (−80°C) until further analysis in the laboratory. In this study, the samples from the 10 , 3, and 0.8 µm filters were analyzed whereas the 0.2 µm Sterivex cartridge filters were analyzed separately (Landa et al., 2016).

### 2.3 DNA and RNA extraction

DNA and RNA were extracted simultaneously from the filters as described previously (West et al., 2016). Briefly, bacterial lysis was achieved by adding to the filters 425 µL lysis buffer (40 mM EDTA, 50 mM Tris, 0.75 M sucrose) and carrying out three freeze‐thaw cycles (liquid nitrogen −65°C) followed by lysozyme treatment (final concentration 1 mg/mL) at 37°C for 45 min. Proteinase K and SDS were added (final concentration 0.2 mg/mL and 1% respectively) to the filters and incubated at 55°C for 1 h. Purification of DNA and RNA was carried out with the Qiagen AllPrep DNA/RNA extraction kit using 1.55 mL RLT + buffer containing β‐mercaptoethanol according to the manufacturer's instructions. The quality of the DNA and RNA was verified by agarose gel electrophoresis and quantified by Picogreen and Ribogreen (Invitrogen) respectively.

### 2.4 cDNA synthesis

cDNA was synthesized from RNA immediately after extraction using the Superscript VILO kit (Invitrogen) according to the manufacturer's instructions using 100 ng RNA.

### 2.5 Illumina sequencing

To determine bacterial diversity and activity, bacterial 16 S rRNA gene fragments of about 450 bp were amplified from 36 DNA samples and 35 cDNA samples (see Table A1) using the primer pair 341F (CCTACGGGNGGCWGCAG) and 805R (GACTACHVGGGTATCTAATCC) described previously (Klindworth et al., 2013). Phytoplankton diversity and activity were assessed by amplifying *rbcL* fragments of 554 bp from 16 surface DNA samples and 4 surface cDNA samples from the 2 bloom stations A3 and F‐L (Table A1) using the Form ID RbcL gene primer set (F: GATGATGARAAYATTAACTC, R: ATTTGDCCACAGTGDATACCA) described previously (Wawrik et al., 2003). The 341F and the *rbcL* forward primers were tagged at the 5ʹ end with different 7 bp tags for each sample, and that were chosen from a set of tags designed to be robust to substitution, deletion and insertion errors incurred in massively parallel sequencing (Faircloth & Glenn, 2012). We also included in parallel a control consisting of DNA from a synthetic mock community (Mock) of 20 bacterial species containing equimolar (even) rRNA operon counts (HM‐782D; Genomic DNA from Microbial Mock Community B, Even, Low Concentration, BEI Resources, Manassas, VA that was amplified as above with the bacterial primer pair. This standard is now obtainable from LGC Standards S.a.r.l.; reference ATCC® MSA‐1002™).

**Table 1:** Brief description of the study sites.

Station	Date (2011)	Z_ML_ (m)	Euphotic layer (m)	Depth (m)	Temp (°C)	Chl a (µg L^−^^1^)^a^	NO_3_^−^+ NO_2_^−^ (µM)^b^	PO_4_^3−^ (µM)^b^	Si(OH)_4_ (µM)^b^	DFe (nM)^c^
R	26/10	105 ± 15	100	20	2.18	0.32	25.71	1.81	12.07	0.088^d^
				60	2.10	0.27	26.22	1.82	12.25	0.076
				150	1.79	0.07	26.64	1.90	14.34	0.181
				300	1.84	b.d	33.45	2.30	38.67	0.33
F	07/11	38 ± 7	35	20	4.31	2.88	19.15	0.91	7.23	0.26
				70	3.19	0.34	27.10	1.93	14.25	0.30
				150	2.52	0.04	29.94	2.01	21.04	n.a
				300	2.88	b.d	33.79	2.26	37.02	0.40
A3	16/11	153 ± 15	38	20	2.25	1.64	25.51	1.75	18.41	0.18^d^
				80	2.16	2.12	26.31	1.79	19.24	0.14
				160	2.15	2.29	26.49	1.81	19.46	n.a
				300	1.91	0.05	33.13	2.25	42.29	0.66
E5	19/11	46 ± 13	55	20	3.26	1.10	25.25	1.71	11.53	0.06
				80	2.98	0.92	26.07	1.83	12.59	0.10
				150	1.94	0.20	28.42	2.00	20.10	0.11
				300	1.94	0.12^d^	34.06	2.36	48.52	0.23

*Note*: Sampling depths and corresponding environmental parameters are given. The mixed layer depth (Z_ML_) is the mean ± sd of all CTD casts performed during the occupation of each station. The Z_ML_ is based on a difference in sigma of 0.02 to the surface value. The euphotic layer is defined as the depth with 1% of surface PAR.

Abbreviations: b.d, below detection; n.a, not available.

^a^ Data are from (Lasbleiz et al. 2014).

^b^ Data are from Blain et al. (2015).

^c^ Data are from Quéroué et al. (2015).

^d^ Values are provided for the depth closest to the sampling depth used for sequence analysis within the Z_ML_ (40 m at R and 37 m at A3‐2).

Before PCR, DNA samples were diluted in molecular grade water (Merck) to a concentration of 10 ng/µL. We decided to pool the 65–10 µm and 25–3 µm DNA and cDNA size fraction samples for depths 20–150/160 m (see Table A1) to give a combined fraction size of >3 µm <65 µm (pooled at equal concentrations of DNA/cDNA). We cannot rule out that phytoplankton falling between 10 and 25 µm may be overestimated due to the overlap between these 2 size fractions. The samples from the 0.8 µm filter corresponded to a fraction size >0.8 µm <3 µm.

DNA/cDNA samples or Mock DNA (1 µL) were amplified in duplicate 10 µL reactions containing 1X KAPA 2 G Fast Ready Mix (Merck) and 0.5 µM of each primer. The PCR cycling conditions were 95°C for 3 min followed by 25 cycles of 95°C for 15 s, 55°C for 15 s and 72°C for 2 s, and a final extension of 72°C for 30 s. Duplicate reactions were pooled and PCR amplification was verified by gel electrophoresis. To normalize the samples before pooling and sequencing, the Sequalprep Normalization Plate (96) kit (Invitrogen) was used according to the manufacturer's instructions. After binding, washing and elution, the environmental and the mock community PCR products were pooled into one tube. The clean‐up of the PCR amplicon pool was achieved with the Wizard SV Gel and PCR Clean‐Up System (Promega) according to the manufacturer's instructions with elution in 30 µL of molecular biology grade water. DNA was quantified with the Quant‐iT PicoGreen dsDNA Assay kit (Invitrogen) according to the manufacturer's instructions. Approximately 700 ng of DNA was pooled with 350 ng of barcoded PCR products from a different project (sequencing the 16S rRNA genes of lichen‐associated bacteria) and sent out to the sequencing company Fasteris for library preparation and sequencing (described below). Library preparation involved ligation on PCR using the TruSeq DNA Sample Preparation Kit (Illumina) according to the manufacturer's instructions except that 5 PCR cycles were used instead of 10 cycles. The library was sequenced on one Illumina MiSeq run using the 2 × 300 bp protocol with MiSeq version 3.0 chemistry and the base‐calling pipeline MiSeq Control Software 2.4.1.3, RTA 1.18.54.0, and CASAVA‐1.8.2. The error rate was measured by spiking the library with about 0.5% of a PhiX library and mapping the reads onto the PhiX reference genome.

## 3 BIOINFORMATIC ANALYSIS

### 3.1 Sequence preprocessing

Paired‐end Illumina sequence reads were initially processed using version 8 or version 9 of the USEARCH‐64 package (Drive5) for read merging, quality filtering, parsing of the reads according to the project and primer removal, with some commands from QIIME version 1.9.1 (Caporaso et al., 2010) and *mothur* version 1.33.3 (Schloss et al., 2009).

Several criteria were chosen in the preprocessing steps to minimize errors and reduce over‐inflation of diversity (Bokulich et al., 2013); (a) only three mismatches were allowed in the overlapping region when merging the paired‐end reads, (b) quality filtering was carried out after merging using a stringent expected error of 1.0 (Edgar & Flyvbjerg, 2015), (c) zero mismatches were allowed in the barcode when demultiplexing, and (d) exact matches to both primers required. More details are found in Appendix 3.

### 3.2 Definition of zOTUs

The UNOISE3 algorithm from the USEARCH‐32 package (version 11) was used to denoise the 16S rRNA or *rbcL* reads (removal of sequencing errors and chimaeras), allowing the recovery of correct biological sequences, also termed zero‐radius OTUs (zOTUs) (see Edgar, 2016) for the description and validation of the previous algorithm UNOISE2).

### 3.3 Classification of 16S rRNA sequences

zOTUs were classified with the USEARCH‐32 v11 classifier SINTAX and the SILVA v123 sequence database using a cutoff of 0.8 (see Appendix 3). zOTUs were aligned using the mothur SILVA SEED alignment (v123) and further filtering was carried out to remove zOTUs unassigned at Kingdom level, or those that were not well aligned. The filtered zOTU table, together with the corresponding taxonomy was converted to biom format for import into the R package Phyloseq (McMurdie & Holmes, 2013) for further sequence manipulation and data exploration.

For bacterial diversity, the PhyloSeq object was filtered to remove Archaea, Eukaryotic, mitochondrial, and plastid sequences.

Of the 232 zOTUs identified as plastids, around 93% were only classified as far as Order level as Chloroplasts using the SILVA 123 database. To obtain a classification at a lower taxonomic level, all zOTUs classified as Chloroplasts were extracted and classified further with the SINTAX classifier as carried out for the bacterial sequences, using the Phytoref Database (Decelle et al., 2015; see Appendix 3) before reimporting into Phyloseq.

### 3.4 Taxonomic assignment of rbcL sequences

In the absence of an rbcL sequence database for marine phytoplankton, zOTU sequences were analyzed by NCBI Blastn (Altschul, 1997) and the hit with the highest percentage similarity including a named species was used to assign the taxonomy with the taxonomic levels retrieved from the WoRMS database (WoRMS Editorial Board, 2016). Sequences with >99% sequence identity to a named species were assigned to species level unless two different species showed the same % identity. In this case, the zOTU was assigned as far as the genus level. zOTUs showing a % ID of 97% or greater were assigned to the genus level. Blastn results with hits <97% were assigned to the lowest taxonomic level possible coherent with the different hits proposed in the results table (e.g., for Zotu74, there were only two hits at 96% with the named species *Teleaulax* sp. and *Phagioselmis* sp, both belonging to the family Geminigeraceae and therefore the zOTU was assigned at this level). In the case of blastn results that retrieved only uncultured sequence hits, even after allowing 250 hits, uncultured clones that had been situated in phylogenetic trees in published data (e.g., Bhadury & Ward, 2009) were used to attempt to assign the sequences at least at the class level.

zOTUS assigned to non‐marine groups including the Phylum Streptophyta (land plants) and the Class Trebouxiphyceae as defined by the World Register of Marine Species (WoRMS) were filtered out of the OTU table as most likely representing cross‐talk OTUs from the lichen project analyzed in the same run.

The *rbcL* sequences were imported into ARB (arb‐6.0), aligned and a neighbor‐joining tree was constructed. The tree was exported in Newick format to be combined with the zOTU table, tax_table, and meta‐data using the Phyloseq package.

### 3.5 Data analysis

Data manipulation and analysis was performed using various packages of the R (version 3.5.1) platform (ape, phyloseq, vegan, mixOmics, ggplot2, dplyr, tidyr, factoextra, dendextend, matrixStats, stringr, reshape2, tidyverse). Alpha diversity metrics were calculated with the phyloseq package and the Shannon Entropy values (x) were transformed to effective species diversities according to (Jost, 2006) by taking the exponential of the values (exp(x)). Rarefaction of zOTU tables was chosen before clustering methods since this normalization method may better reflect the clustering of samples according to their biological origin (Weiss et al., 2017). Nonmetric dimensional scaling (NMDS) plots were generated by Phyloseq using Bray–Curtis dissimilarity (upper three depths) and dendrograms were constructed using hclust() and Ward.D2 clustering. The significance of the bacterial community structure differences represented in the nMDS plots was tested by PERMANOVA using the adonis2 routine in vegan after first verifying the homogeneity of group dispersions with betadisper.

For the figures focusing on specific taxonomic groups (Figures 4, 5, 6, A6 and A7), a reduced data set was used to eliminate potentially spurious bacterial zOTUs from Illumina cross‐talk artefacts. In brief, the variance was calculated for the zOTUs as carried out previously (West et al., 2018), retaining only those zOTUs with a variance >1 × 10^−^^7^. This reduced the zOTU table to 679 taxa but still retained 91.3% ± 2.3% of the total read count when considering the upper three depths of the four stations or 89.7% ± 4.3% when including the 300 m depth samples.

Mantel and partial Mantel tests were performed in vegan using mantel() and mantel.partial() based on the Pearson correlation method. Before correlation analysis, environmental variables (depth, temperature, salinity, oxygen, phosphate, silicic acid, nitrite, nitrate, ammonium, and dissolved organic carbon (DOC)) were *z*‐score transformed and a Euclidean distance matrix was calculated. The bacteria and phytoplankton zOTU matrices underwent Hellinger transformation before calculating Bray–Curtis distance matrices. The correlation heatmaps were generated using spls() and cim() in the mixOmics package with clr‐transformed data (Chun & Keleş, 2010; Rohart et al., 2017).

## 4 RESULTS AND DISCUSSION

### 4.1 Environmental context

Phytoplankton and their associated bacterial communities in the particulate fractions (0.8–3 and >3 µm) were characterized at three stations influenced by Fe fertilization to the north‐east and south‐east the Kerguelen Islands Plateau (stations F, E5, and A3) and at a reference station in the HNLC region west of the plateau (station R) (Figure 1). All stations were located south of the polar front except for station F, and their hydrographic conditions are described in detail in (Park et al., 2014) and (d'Ovidio et al., 2015). Station R was sampled as a reference HNLC site west of the Kerguelen plateau with surface waters characterized by low concentrations of dissolved iron (dFe; <0.088 nM) (Quéroué et al., 2015) and Chl *a* (<0.32 µg L^−^^1^) (Lasbleiz et al., 2016). The three stations subject to natural Fe fertilization had overall higher concentrations of dFe (0.18–0.66 nM) in the wind mixed layer (Z_ML_) and Chl *a* concentration varied considerably, with values ranging from 0.9 (E5) to 4.0 µg L^–^^1^ (F) among the Fe‐fertilized stations. This patchiness in Chl *a* and other biological parameters was due to spatial and temporal variability in the bloom development (Lasbleiz et al., 2016). By contrast, the major inorganic nutrients N and P were similar across sites and characteristic of this region (Blain et al., 2015). Stations R and A3 had deeper Z_ML_ (105 and 153 m, respectively) than stations F and E5 (38 and 46 m, respectively) (Table 1) with the euphotic layer depth (1% surface PAR) roughly equaling the Z_ML_ at all sites except for station A3, where PAR penetrated to only a small fraction of the Z_ML_.

### 4.2 Patterns of bacterial and phytoplankton diversity in size‐fractionated samples

Bacterial and phytoplankton diversity were assessed at the molecular level by generating sequence reads from the 16S rRNA gene (region V3‐V4) and the RubisCO large subunit gene (*rbcL*) respectively from DNA and RNA samples (reverse‐transcribed to cDNA). The *rbcL* gene primers used to target the RubisCO form 1D, present in chromophytic (red) algal groups representing the dominant phytoplankton species in the Southern Ocean (e.g., Wright et al., 2010), but would not target the green algal groups belonging to Chlorophyta and cyanobacteria or dinoflagellates (e.g., Tabita et al., 2008). We chose to use *rbcL* since it is plastid‐encoded and therefore excludes heterotrophic protists that are often overrepresented in 18S rRNA sequence libraries (Georges et al., 2014; Heywood et al., 2011). The *rbcL* gene also performs better than the 18S rRNA gene to distinguish closely related taxa (Evans et al., 2007). In addition, since *rbcL* is a key gene involved in photosynthetic carbon fixation, the analysis of *rbcL* RNA transcripts could be used as a proxy for phytoplankton activity (Wawrik et al., 2003).

**Figure A1:**
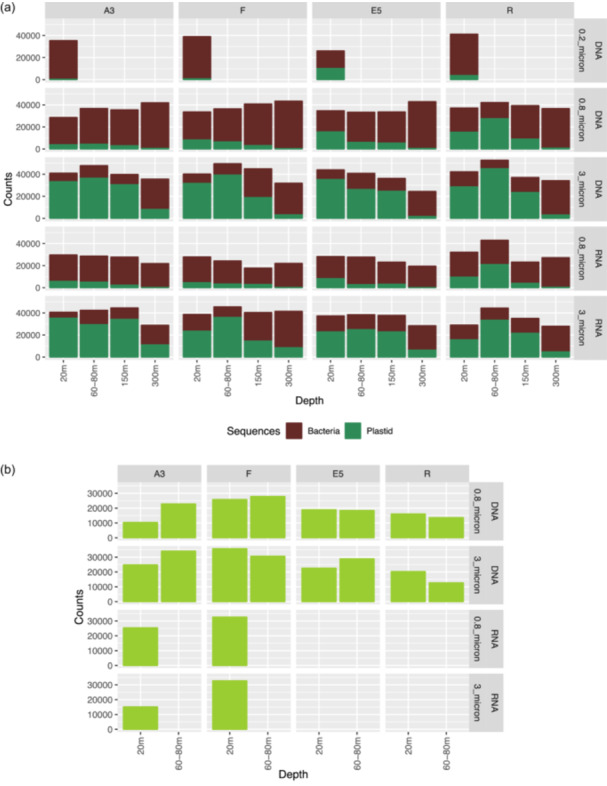
16S rRNA gene (a) and rbcL gene (b) sequencing library sizes obtained from the different sampling sites, depths, and size fractions from DNA and RNA (cDNA). The plastid and bacterial 16S rRNA gene sequences were separated in Phyloseq by subsetting out the Class “Chloroplast,” assigned by the Sintax Silva database.

After paired‐end read joining and quality filtering, 0.9 million 16S rRNA gene reads were denoised followed by clustering into 1719 bacterial zOTUs (100% identity; zero‐radius OTUs) and 232 plastid zOTUs (see Experimental Procedures). Similarly, just over 0.5 million *rbcL* reads were clustered into 222 *rbcL* zOTUs. Although 16S rRNA zOTUs assigned to plastids are often filtered out in bacterial diversity studies, here they were retained as a useful comparison to the picture of phytoplankton diversity revealed by the *rbcL* sequences. The proportional abundance of the 16S rRNA bacterial sequences was highest in the 0.8–3 µm fraction whereas plastid sequences were proportionally more abundant in the >3 µm size fraction except for the 300 m samples where bacterial sequences dominated (Figure A1). To examine the diversity and composition of the active fraction of bacterial and phytoplankton communities, 16S rRNA and *rbcL* gene sequences were also generated from reverse‐transcribed RNA (cDNA) for the majority of the samples for 16S rRNA and from four surface samples at the A3 and F bloom samples for *rbcL* (see Table A1 for details).

The alpha diversity index Shannon Entropy was calculated from rarefied zOTU tables and then transformed to true diversities or “effective species number” for both phytoplankton and bacteria (see Experimental Procedures). This is defined as the number of evenly represented species to give the value of the calculated index which allows an intuitive comparison between the samples (Jost, 2006). For phytoplankton, the 16 S rRNA and *rbcL* data gave diversity estimates of a similar magnitude for a given sample (Table A2). The R and E5 >3 µm size fraction phytoplankton diversity was over threefold higher than that at the bloom stations (A3 and F) whereas the 0.8–3 µm fractions of all stations gave similar true diversities of around 14–20. Similarly, microscopic observations revealed a higher diatom diversity, corresponding mainly to the >3 µm size fraction, at the E5 and R sites as compared to A3 and F (Lasbleiz et al., 2016). Bacterial diversity was generally higher in the >3 µm fraction than in the 0.8–3 µm fraction in the two surface depths. When ranks of bacterial diversity and depth were compared (1–4, lowest to highest diversity or surface to deep; Table 2), diversity was strongly positively correlated with depth (*R* ^2^ = 1) for the 0.8–3 µm fraction, while for the >3 µm fraction there was no significant correlation between diversity and depth.

**Table 2:** Impact of different factors on bacterial community structure tested by PERMANOVA analysis.

Factor	Station	Fraction	Depth (m)	Sample type	Residuals
Values	A3,F,E5,R	0.8 µm, >3 µm	20,60–80,150	DNA, RNA	
DNA(*n* = 24), RNA (*n* = 24)	0.24 (0.001)	0.14 (0.001)	0.11 (0.001)	0.09 (0.001)	0.42

*Note*: *R* values are indicated with the *p* Value in brackets.

### 4.3 Distinct phytoplankton community structures at the different stations

**Figure 2:**
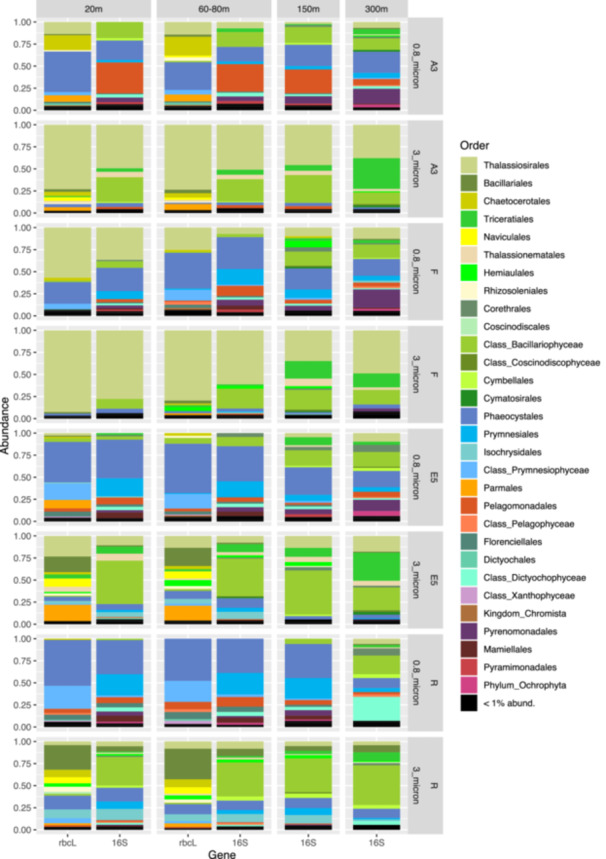
Comparison of phytoplankton taxonomic community structure (order level) at different depths at the four sampling stations in the 0.8–3 µm or >3 µm size fractions as revealed by plastid rbcL and 16S rRNA marker gene sequencing. Diatom orders are shown in shades of green, yellow, and beige from Thalassiosirales to Cymbellales.

**Figure A2:**
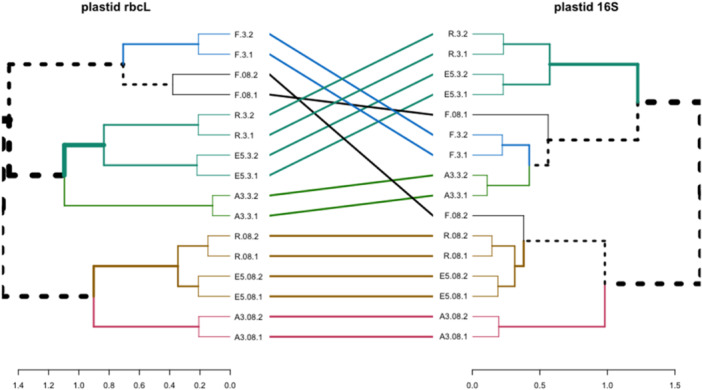
Phytoplankton community structure comparisons in the top two surface depths 20 m (1) and 60–80 m (2) at stations A3, F, E5, and R, recovered from two size fractions 0.8–3 µm (0.8) and >3 µm (3) assessed by plastid 16S rRNA and rbcL marker gene sequencing. Dissimilarity dendrograms using Bray–Curtis distances were constructed for each marker gene and their level of congruence was examined by Tanglegram. Sample notation is the Station Name.SizeFraction.Depth.

Phytoplankton communities clustered by a fraction and by station for the upper two surface depths (20–80 m) for both phytoplankton marker genes which showed good agreement apart from the samples from station F (see tanglegram, Figure A2). For both fractions, the communities at R and E5 were most closely related to each other and fell into a cluster with the communities from A3. Whereas the *rbcL* sequences grouped the 0.8–3 and >3 µm fractions in a separate cluster, the 16S rRNA sequences grouped the A3 and F >3 µm samples with one of the 0.8–3 µm fraction samples. The difference in clustering may be explained by the higher diversity of the *rbcL* sequences compared to the 16S rRNA sequences which allowed the differentiation of two distinct *Thalassiosira* sp. zOTUs at A3 and F whereas a single common zOTU was detected in the 16S rRNA data set (Figure 2, discussed below).

**Figure A3:**
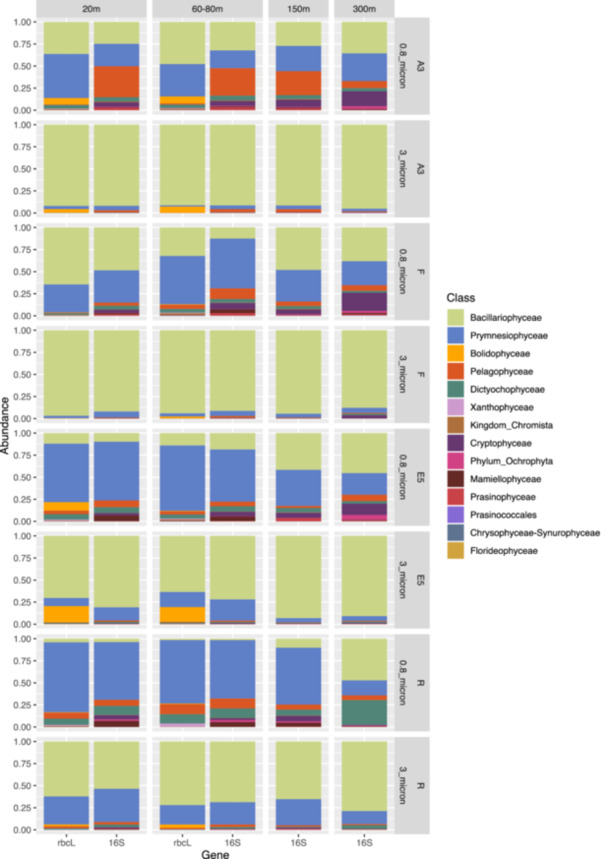
Comparison of phytoplankton taxonomic community structure (class level) at different depths at the four sampling stations in the 0.8–3 µm or >3 µm size fractions as revealed by plastid rbcL and 16S rRNA marker gene sequencing.

In the >3 µm fraction of the upper two surface depths, Bacillariophyceae accounted for >90% of the sequences at stations A3 and F but exhibited lower relative abundances (>60%) in the same size fraction at the E5 and R stations (Figure A3). The lower relative abundance of diatoms at E5 and R compared to A3 and F was also observed by (Lasbleiz et al., 2016) who assessed the abundance of different phytoplankton groups by microscopy counts and flow cytometry. Diatoms accounted for 72%–74% of the phytoplankton groups in the euphotic zone at A3 and F but only 38% and 7% respectively at E5 and R (Lasbleiz et al., 2016). Conversely, in the 0.8–3 µm fraction Prymnesiophyceae largely dominated the R and E5 sequences in the present study. Although Prymnesiophyceae were the most abundant class in the majority of the 0.8–3 µm fraction samples of the bloom stations, attaining >50% of sequences at 80 m at F and 20 m at A3, Bacillariophyceae sequences remained relatively abundant even in this smaller size fraction. At the two surface depths for which *rbcL* and 16S rRNA plastid sequences were available for comparison (20–80 m, Table A1), the two analyses yielded consistent relative abundances of the two major classes Bacillariophyceae (diatoms) and Prymnesiophyceae (haptophytes). Notable differences between the two marker genes included the higher relative abundance of Pelagophyceae and Cryptophyceae zOTUs in the 16S rRNA data set compared to the *rbcL* data set, whereas the silica‐containing Bolidophyceae group that is closely related to diatoms was observed in the *rbcL* but not the 16S rRNA data set. It is not uncommon to see differences in the relative abundance of phytoplankton groups when using different primer pairs due to the effects of primer bias as observed by (Shi et al., 2011). In their study, Pelagophyceae and Dictophyceae sequences were favored with one pair whereas Prymnesiophyceae dominated the sequences obtained with the second pair, underlining the utility of multiple marker gene comparisons to obtain a fuller picture of phytoplankton diversity.

**Figure 3:**
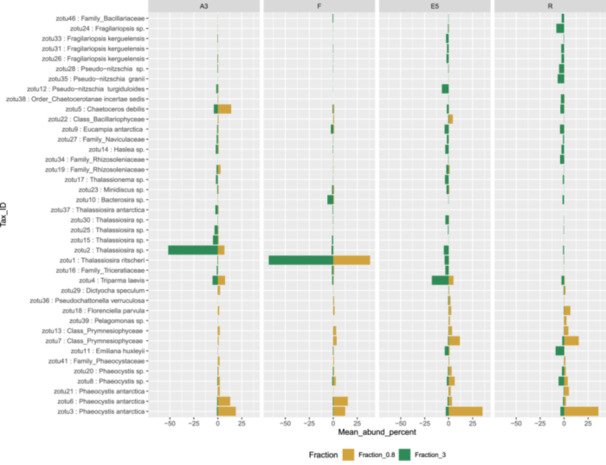
Mirror column chart to compare the relative mean abundance (upper two surface depths) of the 40 most abundant rbcL zOTUs in the 0.8–3 µm and >3 µm fractions at the four stations.

At the Order level, in the >3 µm fraction, both marker genes indicated a dominance of Thalassiosirales diatoms in the bloom stations A3 (73% of total sequences) and particularly at F, where these sequences accounted for >90% of the total *rbcL* sequences (Figure 2). Surprisingly, at A3, higher relative abundances of the diatom order Chaetocerotales were observed in the *rbcL* sequences of the smaller 0.8–3 µm fraction compared to the >3 µm fraction whereas the converse was true at R. For the 16S rRNA sequences, the A3 and F stations shared the same main zOTU that was assigned at Order level (Thalassiosirales; data not shown) whereas the *rbcL* sequences distinguished the dominant zOTU at these two stations (Figure 3). A single *rbcL* zOTU assigned as *Thalassiosira ritscheri* accounted for 60%–75% of the sequences at F and less than 0.4% at A3, whereas the second most dominant zOTU was assigned as *Thalassiosira* sp. (97% similarity to *Thalassiosira antarctica*) and accounted for 49%–54% of the sequences at A3 and less than 2.1% at F. The >3 µm phytoplankton communities at the HNLC station R and E5 showed a more diverse and even distribution with higher relative abundances of *Fragilariopsis* spp, *Pseudo‐nitzschia* spp., *Chaetoceros* spp. and *Eucampia* spp. The haptophyte *rbcL* sequences recovered from the 0.8–3 µm fraction were dominated by two roughly equally abundant *Phaeocystis* zOTUs at the bloom stations A3 and F (zOTU3 and zOTU6) and a single *Phaeocystis antarctica* (zOTU3) at E5 and R. The major *Thalassiosira* zOTU1 also accounted for a significant fraction of the *rbcL* sequences in the 0.8–3 µm fraction at F (Figure 3).

We compared the relative abundance of diatoms as assessed by *rbcL* gene sequencing from the >3 µm samples to microscopy counts carried out on CTD rosette samples (Lasbleiz et al., 2016 and Table A3). To allow comparisons, the species identified by microscopy and the *rbcL* zOTUs were grouped, when possible, at the order level, apart from a few low‐abundance species that did not fall into the orders listed and were grouped instead under “Class_Bacillariophyceae.” The main differences were observed for the Thalassiosirales and Chaetocerotales at the bloom stations where our *rbcL* sequence data set appeared to overestimate Thalassiosirales abundance: in particular the dominance of *Thalassiosira* spp. at A3 diverges from the microscopy counts where *Chaetoceros* (Hyalochaete) spp. accounted for 60% and 80% of carbon biomass and live cell abundance respectively (Lasbleiz et al., 2016, Table A3). This could be explained by our 65 µm prefiltering step that could have reduced the number of the *Chaetoceros* chain‐forming diatoms in the sequence datasets or less efficient lysis of certain species with highly silicified frustules at the nucleic extraction step (Luddington et al., 2016). For station R, the diatom order abundances were relatively similar between microscopy counts and sequencing data except for the absence of Thalassionematales in the *rbcL* data.

**Figure A4:**
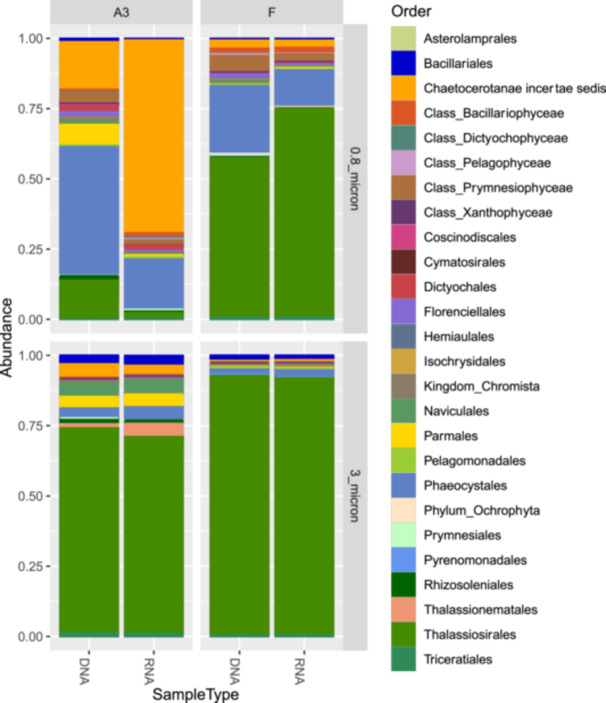
Comparison of presence and activity of phytoplankton (order level) by rbcL gene sequencing from DNA or RNA (cDNA) extracted from the 0.8–3 µm or >3 µm fractions collected at 20 m at the bloom stations A3 and F.

The analysis of the comparatively small RNA (cDNA) *rbcL* data set obtained from the surface depth of 20 m at the bloom stations A3 and F provided further evidence that the dominant diatoms affiliated to *Thalassiosira* spp. were also active, given the similar relative abundances in the >3 µm fraction for RNA or DNA. Interestingly the 0.8–3 µm size fraction showed more pronounced differences between the RNA and DNA data sets, notably at station A3. Whereas *Thalassiosira* sequences were also the major contributor to the station F RNA sequences, at A3, *Chaetoceros* spp. accounted for >65% of the RNA sequences (Figure A4).

Therefore, despite the apparent overestimation of *Thalassiosira* spp. in the *rbcL* DNA sequence data, we were nonetheless able to detect the main diatom species and highlight a dominant Chaetocerales activity in the RNA data set at A3 in agreement with (Lasbleiz et al., 2016). Furthermore, we were also able to assess the diversity and activity of the smaller haptophyte phytoplankton that are difficult to identify by light microscopy. The evaluation of protist diversity at the KEOPS2 stations by 18S rRNA gene sequencing (Georges et al., 2014) revealed the presence of small diatom species at the bloom stations such as *Thalassiosira* sp., *Minidiscus* sp. and *Coscinodiscus* sp. but failed to detect certain species such as *Fragilariopsis* sp. 18S rRNA Haptophyta sequences were also dominated by *Phaeocystis antarctica* in agreement with our *rbcL* and 16S rRNA data. However, Bacillariophyceae were underestimated seemingly due to the overrepresentation of alveolates that possess a high rRNA operon copy number (Zhu et al., 2005) and that are known to dominate 18S rRNA gene metabarcode databases (Vaulot et al., 2022).

### 4.4 Bacterial community composition

**Figure A5:**
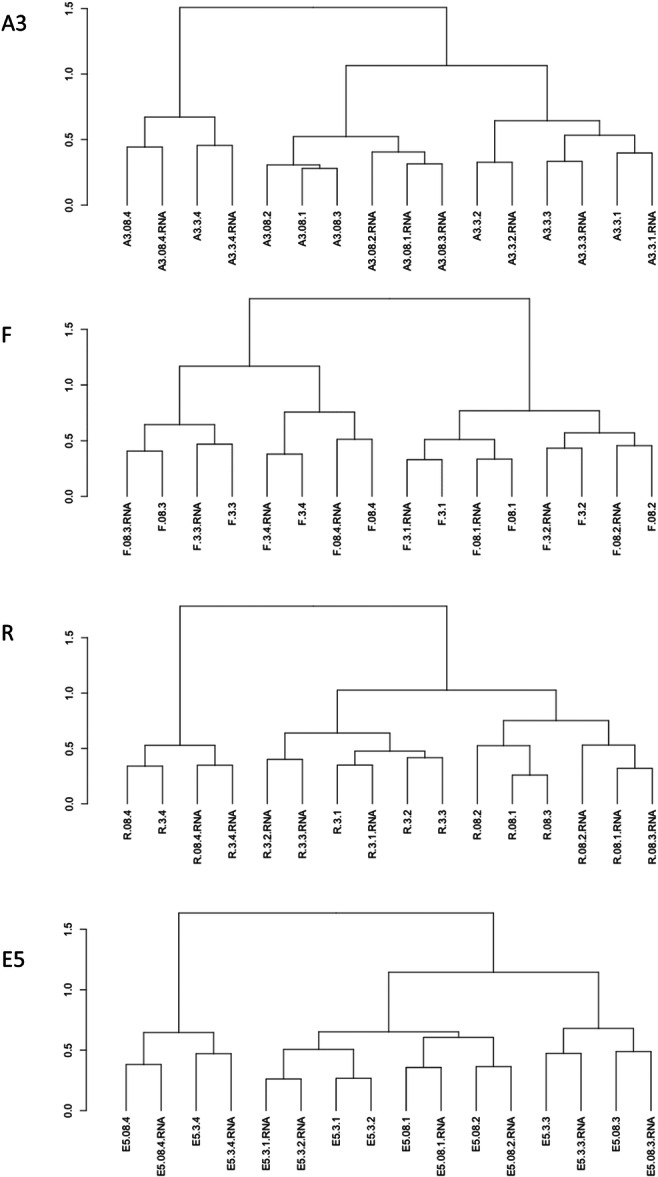
Bacterial community structure comparisons at depths 20 (1), 60–80 (2), 150 (3), and 300 m (4) at stations A3, F, E5, and R, recovered from two size fractions 0.8–3 µm (0.8) and >3 µm (3) assessed by 16S rRNA gene sequencing from DNA and RNA (RNA). Dissimilarity dendrograms were constructed using Bray–Curtis distances. The sample notation is StationName.SizeFraction.Depth for DNA samples and Name.SizeFraction. Depth.RNA for RNA samples.

Beta diversity analysis on the rarefied bacterial 16S rRNA gene (DNA and cDNA) sequences revealed that for the bloom stations A3 and F, the community composition was similar between the DNA and RNA (cDNA) samples except for the A3 0.8–3 µm fraction samples at the three depths from 20 to 150 m where the DNA samples clustered separately from the RNA samples. The converse was observed at station R where all DNA and RNA samples clustered separately apart from the 20 m >3 µm sample (Figure A5). Community structure comparisons of marker gene sequences originating from RNA and DNA to reveal activity (protein synthesizing potential; Blazewicz et al., 2013) and presence, respectively, have been applied to marine and freshwater bacterial communities (Denef et al., 2016; Hunt et al., 2013). 16S rRNA gene sequences from RNA and DNA were also compared with metatranscriptomic analysis on the samples to investigate the impact of a phytoplankton bloom on bacterial diversity (Wemheuer et al., 2014). The high coupling between the composition of the total (DNA) and the active (RNA) bacterial communities observed only in bloom stations suggests a stronger role of bottom‐up control in the shaping of community composition in response to an increased supply of photosynthate. In contrast, higher fractions of dormant cells and slow‐growing bacteria could further explain the distinct clustering between the total and active bacterial communities at the HNLC site.

**Figure 4:**
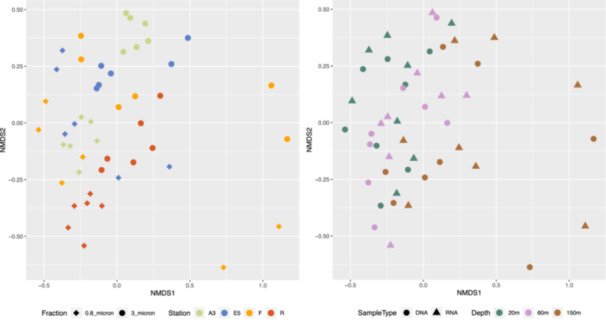
The similarity of the bacterial communities in different size fractions collected from the four stations in three depth layers (20, 60–80, and 150 m) assessed by 16S rRNA marker gene analysis of DNA or RNA (cDNA) using Bray–Curtis distances and nMDS. The same nMDS is shown in two separate panels to show the separation of the samples by fraction and station (left) and by depth (right). Size fraction 0.8_micron (0.8–3 µm), 3_micron (>3 µm).

**Figure 5:**
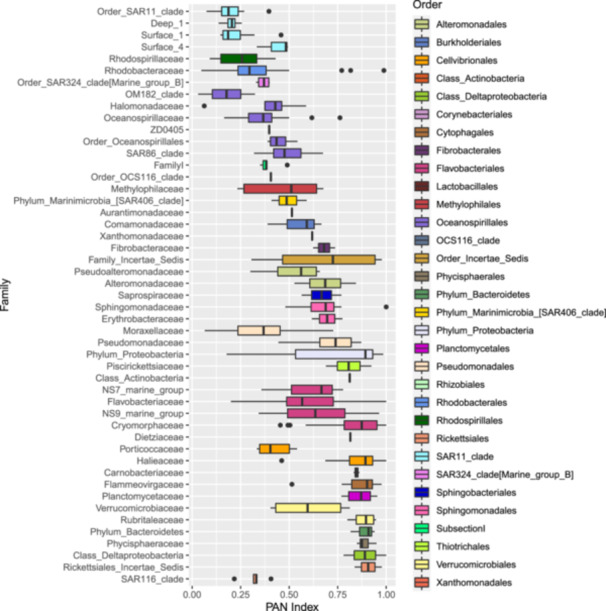
The size fraction preference of zOTUs in the mixed layer depths (Z_ML_) at the four stations inferred by calculating a Particle associated Niche Index (PAN index) and illustrated in a boxplot with zOTUs grouped at the family level. zOTUs with a PAN index close to zero are prevalent in the 0.8–3 µm fraction whereas those with a PAN index toward one are more abundant in the >3 µm fraction. Subsection I, Family I corresponds to *Synechococcus* sp., Family Incertae Sedis to *Marinicella* sp. and the family Dietziaceae is affiliated to the Corynebacteriales order. Families are color‐coded according to order as illustrated in the legend.

**Figure A6:**
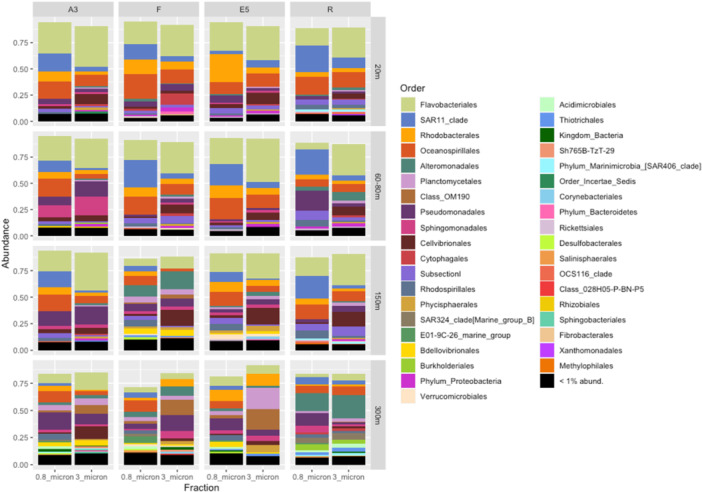
Bacterial taxonomic diversity at the order level (DNA) at different depths for the four sampling stations in the 0.8–3 µm (0.8_micron) or >3 µm (3_micron) size fractions.

Bacterial community composition was significantly different between sample stations, size fractions, sample type and depth, as illustrated in the nMDS plots in Figure 4 and supported by the PERMANOVA analysis in Table 2. These differences can also be observed by the relative abundance of the different taxonomic groups in Figure A6 and the hierarchical clustering shown in Figure A5. Communities in both size fractions from below the Z_ML_ clustered separately from those within the Z_ML_ and this was particularly noticeable at station F where the Z_ML_ was relatively shallow (See Z_ML_ in Table 1 and Figure A5). At a glance, even at the order level, there were clear differences in the taxonomic composition of the different size fractions. Our results are in line with studies from other marine environments showing that particles of different sizes harbor distinct bacterial communities, and that specific bacterial taxa are found preferentially associated with certain particle sizes (Mestre et al., 2017, 2020; Yung et al., 2016). To reveal differences in the preferences of different bacterial taxa for the particulate niches represented by the two size fractions, a Particle‐Association Niche Index (PAN‐Index) was calculated for each zOTU as described previously (Mestre et al., 2018; Salazar et al., 2015). The PAN‐Index of taxa found predominantly in the 0.8–3 µm fraction showed a value towards 0 whereas taxa found more often in the >3 µm fraction had a value closer to 1 (Figure 5).

In the mixed layer depths (Z_ML_), the >3 µm particle niche was characterized by several Proteobacteria families including Alteromonadaceae, Colwelliaceae, Pseudomonadaceae, Halieaceae, Bdellovibrionaceae, Erythrobacteraceae, and Sphingomonadaceae. All the families belonging to the Bacteroidetes phylum were preferentially associated with this larger particle fraction size, as were the Planctomycete families Phycisphaeraceae and Planctomycetaceae and those belonging to the Verrucomicrobia. Low PAN index clades comprised several Oceanospirillales families including SAR86, different SAR11 clades, Moraxellaceae, Marine group B (SAR324), Porticoccaceae (SAR92), and the families Rhodospirillalaceae and Rhodobacteraceae. *Synechococcus* zOTUs were also most abundant in the 0.8–3 µm fraction as observed previously (Mestre et al., 2017), most likely due to their larger cell size than heterotrophic bacteria. However, relatively high abundances (5.5%–6%) were also observed in the >3 µm fractions at stations R and F, respectively, which could be explained by the formation of aggregates, attachment to particles or due to symbiotic relationships. *Synechococcus*‐like symbionts have been detected in dinoflagellates, tintinnids and radiolarians (Foster et al., 2006) and in Foraminifera (Bird et al., 2017). Furthermore, high abundances of *Synechococcus psbO* marker gene sequences were also recovered in metagenomic libraries from large‐size fractions collected by Tara Oceans (Pierella Karlusich et al., 2023).

### 4.5 Abiotic versus biotic variables influencing bacterial community structure

To explore the influence of abiotic versus biotic variables on the bacterial community structure, partial Mantel tests were performed between dissimilarity matrices of bacterial 16S rRNA gene zOTUs versus phytoplankton zOTUs assessed either by plastid 16S rRNA gene or by *rbcL* gene diversity (biotic) and versus an environmental variable matrix (abiotic). In the upper two surface depths (20 m and 60–80 m) bacterial communities were significantly correlated with phytoplankton (based on 16S rRNA) for both size fractions, but not to environmental variables (Table A4). When all depth layers were considered, bacterial communities were correlated with phytoplankton but more strongly with environmental variables, highlighting the impact of the environmental conditions set by depth on bacterial community composition. For the *rbcL* data, only the 20 m and 60–80 m surface samples were available, and for both size fractions, bacterial communities showed significant correlations with phytoplankton but not with environmental variables. These observations are in line with the conclusions from several recent studies showing that biotic interactions could be more important for structuring plankton community composition than abiotic factors (Arandia‐Gorostidi et al., 2022; Lima‐Mendez et al., 2015; Liu et al., 2019; Needham et al., 2018).

### 4.6 Phytoplankton‐bacteria co‐occurrences

Given the strong correlation between the phytoplankton and bacterial communities indicated by the partial Mantel tests, we identified specific correlations between the most relatively abundant phytoplankton and bacterial zOTUs (representing >1% of the total sequences in at least one sample) for the upper two surface depths (20 m and 60–80 m) by sparse partial least squares (PLS) regression provided by the MixOmics R package. Correlations were done between the *rbcL* zOTUs and 16S rRNA bacterial zOTUs from DNA samples (Figure 6a) and also between the 16S rRNA plastid and bacterial zOTUs from DNA and from RNA (cDNA) potentially representing the active zOTU interactions (Figure A7). Further information on the relative abundance of the strongly correlating bacterial zOTUs and their potential activity is presented below the heatmap (6b).

Figure 6(a) Clustered image heatmap showing the most significant correlations between the surface bacterial 16S rRNA zOTUs and phytoplankton rbcL zOTUs (upper 2 surface depths). The main bacterial zOTU clusters are outlined in blue and illustrated similarly in (b). (b) For clarity, the summed relative abundances (DNA) of bacterial surface zOTUs in the 2 size fractions (0.8–3 and >3 µm fraction) and their relative activity (ratio of RNA:DNA relative abundance) is shown only for the two main bloom stations A3 and F (omitting the meander station E5) for comparison with the HNLC station R.

**Figure:**
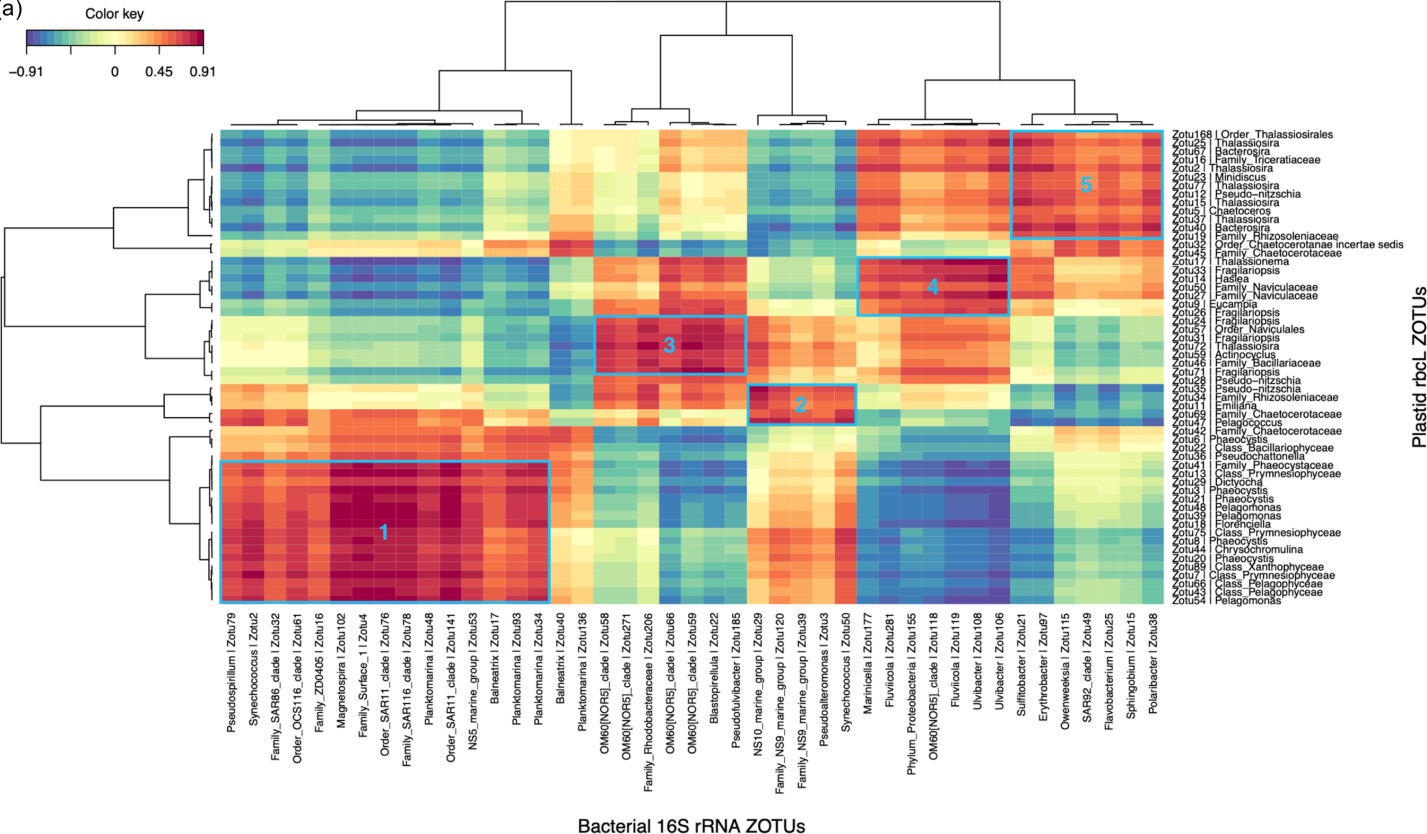

**Figure:**
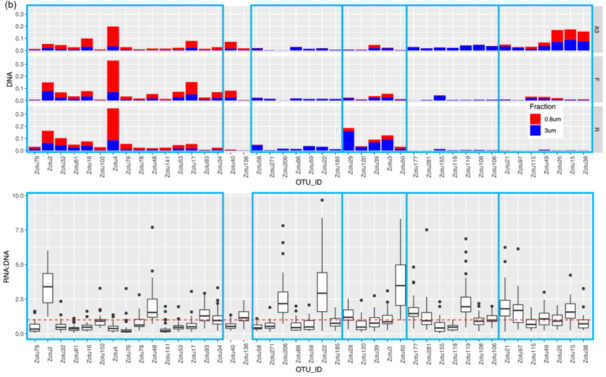

This correlation analysis presented in Figure 6a identified 56 and 43 strongly positively correlated (*r* > 0.6) phytoplankton and bacterial zOTUs, respectively. Together, the phytoplankton zOTUs correlating to bacterial zOTUs contributed from 59.8% to 89.9% (*n* = 12) of total phytoplankton communities at the two surface depths when excluding station F, for which the relative abundances of correlating zOTUs was significantly lower (8.9% to 63.7%, *n* = 4), in particular in the >3 µm fraction (Table A5). In contrast, the contribution of bacterial zOTUs correlating to phytoplankton was lower but more stable (31.0%–49.9% *n* = 16) with no particular patterns between stations (Table A5). The difference between phytoplankton and bacteria in the contribution of correlated zOTUs to total communities could reflect a general tendency of phytoplankton to associate with specific bacteria while from the bacterial point of view, a significant proportion of the community could behave like generalists and thrive on particles, irrespective of the identity of phytoplankton present within the particles. This could suggest that the bacterial zOTUs identified as correlated to phytoplankton zOTUs could be involved in true partnerships with specific phytoplankton taxa.

Bacterial and phytoplankton zOTUs with strong positive correlations (*r* > 0.6) were distributed in several distinct clusters driven in part by the compositional differences observed between the two size fractions (Figure 6a). Cluster 1 contained taxa associated with the 0.8–3 µm fraction with the strongest correlations between the Prymnesiophyceae and Pelagophyceae phytoplankton zOTUS with SAR11, SAR116, and OCS116 clades, *Magnetospira* and 3 *Planktomarina* (Roseobacter RCA clade) zOTUS. These latter zOTUs (zOTU48, 93, 34) were the only members of the cluster showing a higher relative activity as observed in Figure 6b (excluding *Synechococcus*). In this cluster, the presence of typical pelagic oligotrophs such as SAR11 and the Roseobacter RCA clade (*Planktomarina*) and their high correlations with Pelagophyte (*Pelagomonas* sp.) and Haptophyte (*Phaeocystis* sp.) zOTUs suggest that for some of these bacterial taxa, interactions with phytoplankton might be more prevalent than previously recognized. Such interactions could rely on the exchange of organic sulfur compounds such as DMSP or sulfonates, which prymnesiophytes are known to produce, that could fulfill the organic sulfur requirements of taxa that have lost the sulphate assimilation pathway such as the SAR11 (Durham et al., 2019; Tripp et al., 2008). Our data suggest that physical association, either transient or more stable, could play a part in the nutrient exchange between these organisms, as was also suggested by a few other studies that identified SAR11 ecotypes in >0.8 µm size fractions (Allen et al., 2012; Mestre et al., 2017). Our findings differ from those of a *Phaeocystis‐dominated* bloom in an Antarctic polyna, where the *Phaeocystis* cells were in a colony form, and consequently fell into the larger >3 µm fraction, correlating instead with the Gammaproteobacteria SAR92 (Delmont et al., 2014). In our study, SAR92 was most abundant at A3 but did not exceed more than 2.8% of the total bacterial community and did not show a particular size fraction preference (Figure 5b, Porticoccaceae), although the correlation with one SAR92 zOTU was strongest with diatom zOTUs (Figure 6, cluster 5). In a previous study, samples were taken during the peak and decline of the diatom bloom at A3, and SAR92 was both relatively abundant and active in the surface waters (Obernosterer et al., 2011; West et al., 2008), supporting the correlation observed in this study.

Cluster 5 highlighted correlations between several *Thalassiosira*, *Pseudo‐nitzschia*, *Bacterosira,* and *Chaetoceros* zOTUs, with two Roseobacter zOTUs (*Sulfitobacteria* and *Erythrobacter*), Bacteroidetes zOTUs (*Polaribacter*, *Flavobacterium* and *Owenweeksia*), SAR92, and *Sphingobium.* These zOTUs were generally more abundant at A3 than F or R and also showed higher relative activity, particularly for the Roseobacters and *Sphingobium* (Figure 6b). *Sphingobium* is often described as a polycyclic aromatic hydrocarbon degrader but reports of the interactions of this taxon with phytoplankton are scarce and would warrant further investigation. Roseobacter OTUs were also amongst the most active members of the bacterial communities dominating the peak of the bloom at the same station A3 sampled during the KEOPS1 study (Obernosterer et al., 2011; West et al., 2008). Associations between diatoms and members of the highly diverse Roseobacter clade are among the best‐characterised marine microbial partnerships that have important repercussions for marine biogeochemical cycles (e.g., Buchan et al., 2014). Many phytoplankton are vitamin B_12_ auxotrophs (Croft et al., 2005) and therefore these associations typically revolve around the trading of essential metabolic currencies, such as the provision of the sulfonates in exchange for vitamin B_12_, known to be synthesized by many Roseobacter clade members (Durham et al., 2015). There are several reports of the specific association of *Sulfitobacter* strains with *Pseudo‐nitzschia* species that promote the growth of the diatoms through their provision of B vitamins (Andrew et al., 2022) or by the secretion of the growth‐promoting hormone indole‐3‐acetic acid (Amin et al., 2015). An extensive study of Roseobacter biogeography from an Australian time series revealed the co‐occurrence of *Planktomarina* and *Sulfitobacter* with high DMSP‐producing phytoplankton, such as *Phaeocystis* (O'Brien et al., 2022). Although also correlated to *Thalassiosira*, one *Sulfitobacter* amplicon sequence variant (ASV) showed the highest correlations to *Phaeocystis* that exceeded those of SAR11 and SAR86 (O'Brien et al., 2022). This is in contrast to our size fractionated data where the *Planktomarina* and *Sulfitobacteria* were significantly correlated to *Phaeocystis* and diatoms respectively.

Cluster 4 implied potential interactions between *Thalassionema*, *Fragilariopsis*, *Eucampia,* and Naviculaceae with several Flavobacteria zOTUs (*Ulvibacter* and *Fluviicola*) and the Gammaproteobacteria *Marinicella* and OM60[NOR5]. Clusters 2 and 3 revealed correlations between the NS9 and NS10 bacteroidetes groups and *Pseudoalteromonas* with both haptophytes and diatoms (Cluster 2) and between the Gammaproteobacteria OM60[NOR5], the Bacteroidetes *Pseudofulvibacter* zOTU and the Planctomycete *Blastopirellula* with diatom zOTUs (Cluster 3).

For bacterial zOTUs preferentially found in the >3 µm size fraction and correlating to diatom zOTUs, the majority belonged to the phylogenetic groups Flavobacteria, Gammaproteobacteria, and Roseobacter that are typically found in association with phytoplankton (Abell & Bowman, 2005; Liu et al., 2019; Tran et al., 2023). The distinct associations observed for specific diatoms could result from substrate preferences that vary among bacterial taxa (Bunse et al., 2021; Krüger et al., 2019; Xing et al., 2015) responding to differences in the composition of organic matter released by diatom species (Mühlenbruch et al., 2018). Genomic analyses of Flavobacteria demonstrate that they possess many genes involved in adhesion and gliding motility as well as a unique secretion system (Fernández‐Gómez et al., 2013; Gavriilidou et al., 2020) that allows them to employ their diverse repertoire of enzymatic capabilities for the degradation of complex organic matter (Kappelmann et al., 2019). These traits are thought to facilitate a particle‐attached lifestyle, including physical association with phytoplankton. Our observation of several Flavobacteria members with relative abundances correlating with those of several diatoms in the >3 µm size fraction supports the idea that members of this group thrive in the phycosphere and further suggests specific interactions with phytoplankton.

In conclusion, our combined bacterial 16S rRNA and plastid gene metabarcoding approach on size‐fractionated samples from DNA and RNA extracts revealed new insights into potential bacteria‐phytoplankton associations that could pave the way for further laboratory or in situ studies. In contrast to the 18S rRNA marker gene, the 16S rRNA and rbcL plastid marker genes targeted directly the phytoplankton and gave a complementary picture of the diversity. Furthermore, the more variable *rbc*L gene was a more informative marker, allowing the discrimination of the dominant phytoplankton species between the two main bloom stations. In analyzing size‐fractionated samples, we were able to reveal distinct groups of bacterial taxa preferentially co‐occurring with the different phytoplankton size classes dominated by *Phaeocystis* spp. and diatoms, two phytoplankton groups with key ecological and biogeochemical roles. By including metabarcoding sequencing from bacterial 16S rRNA and phytoplankton rbcL transcripts, we were able to add a new dimension to our data by revealing a potential elevated relative activity (RNA:DNA > 1) of all highly correlated Roseobacter zOTUs, *Sphingobium*, and the *Thalassiosira* and *Chaetoceros* zOTUs. The function and importance of the potential interactions we revealed could be investigated in more depth by combining metagenomic and metatranscriptomic analyses of key phytoplankton‐bacteria partners in co‐culture experiments and natural samples during bloom transitions from diatoms to *Phaeocystis*.

## AUTHOR CONTRIBUTIONS

**Nyree J. West**: Conceptualization (supporting); data curation (lead); formal analysis (lead); investigation (equal); methodology (equal); writing—original draft (lead); writing—review & editing (equal). **Marine Landa**: Investigation (equal); methodology (equal), writing—review & editing (equal). **Ingrid Obernosterer**: Conceptualization (lead); funding acquisition (equal); investigation (equal); methodology (equal); project administration (lead), resources (equal); writing—review & editing (equal).

## CONFLICT OF INTEREST STATEMENT

The authors declare no conflict of interest.

## ETHICS STATEMENT

None required.

## Data Availability

Sequence data are openly available in the European Nucleotide Archive under accession number PRJEB23734: https://www.ebi.ac.uk/ena/browser/view/PRJEB23734.

## APPENDIX 1

**Table A1:** Description of the samples analyzed at the four stations in terms of sampling depths and size fractions for 16S rRNA marker gene sequencing from DNA and RNA (cDNA).

Station	Depths (m)	Fractions (µm) for DNA sequencing			Fractions (µm) for cDNA sequencing		
A3‐2	20	0.2–0.8	0.8–3	3–65	0.2–0.8	0.8–3	10–65^a^
	80	na	0.8–3	3–65	na	0.8–3	3–65
	150	na	0.8–3	3–65	na	0.8–3	3–65
	300	na	0.8–3	3–25	na	0.8–3	3–25
F	20	0.2–0.8	0.8–3	3–65	0.2–0.8	0.8–3	3–65
	70	na	0.8–3	3–65	na	0.8–3	3–65
	150	na	0.8–3	3–65	na	0.8–3	3–65
	300	na	0.8–3	3–25	na	0.8–3	3–25
E5	20	0.2–0.8	0.8–3	3–65	0.2–0.8	0.8–3	3–65
	80	na	0.8–3	3–65	na	0.8–3	3–65
	150	na	0.8–3	3–65	na	0.8–3	3–65
	300	na	0.8–3	3–25	na	0.8–3	3–25
R	20	0.2–0.8	0.8–3	3–65	na	0.8–3	3–65
	60	na	0.8–3	3–65	na	0.8–3	3–65
	150	na	0.8–3	3–65	na	0.8–3	3–65
	300	na	0.8–3	3–25	na	0.8–3	3–25

*Note*: rbcL sequences were also analyzed for those samples highlighted in green.

Abbreviation: na, not analyzed.

^a^ The RNA extraction from the 3 µm filter failed.

**Table A2:** Alpha diversity of phytoplankton and bacterial communities in surface depths at the different stations converted into true diversities or “effective species number” (Jost, 2006) from the Shannon Index calculations (DNA).

Station	Depth	Fraction	*rbcL* (phytoplankton plastids)	16S rRNA (phytoplankton plastids)	16S rRNA (bacteria)
A3	20	0.8–3 µm	20.69	12.80	115.18
	60–80		26.94	21.60	127.19
	150			18.06	136.14
	300			37.62	394.36
	20	>3 µm	12.21	13.05	159.09
	60–80		9.27	14.83	142.21
	150			14.09	130.03
	300			7.88	286.17
F	20	0.8–3 µm	7.74	16.46	90.35
	60–80		20.70	25.34	113.78
	150			34.44	317.58
	300			39.12	375.17
	20	>3 µm	3.95	4.94	113.99
	60–80		8.00	11.35	177.57
	150			14.95	180.07
	300			14.91	148.19
E5	20	0.8–3 µm	17.19	19.30	80.09
	60–80		16.33	22.36	101.26
	150			30.86	199.08
	300			44.65	354.15
	20	>3 µm	33.90	31.65	148.15
	60–80		36.28	37.19	138.95
	150			30.15	166.93
	300			20.02	115.55
R	20	0.8–3 µm	14.68	21.16	131.80
	60–80		14.13	15.90	117.70
	150			23.03	154.18
	300			28.33	164.07
	20	>3 µm	39.61	44.19	168.96
	60–80		38.48	32.30	151.75
	150			41.51	142.69
	300			38.19	171.77

**Table A3:** Relative abundances of diatoms (primarily order level) at the four stations obtained by microscopy identification (Lasbleiz et al., 2016) of CTD rosette surface samples, compared to those obtained in our >3 µm size fraction samples from 20 m using rbcL marker gene sequencing.

Order	A3‐20m	A3‐12m	F‐20m	F‐25m	E5‐20m	E5‐17m	R‐20m	R‐28m
	rbcL	Counts	rbcL	Counts	rbcL	Counts	rbcL	Counts
Bacillariales	3.4	3.4	1.9	8.1	25.1	22.3	45.1	43.2
Chaetocerotales	5.2	87.5	0.0	26.1	3.8	37.8	13.6	7.8
Class_Bacillariophyceae	0.0	0.0	0.0	0.0	1.7	0.3	3.2	0.2
Coscinodiscales	0.0	0.0	0.0	0.0	0.0	0.0	3.8	0.0
Hemiaulales	0.0	0.0	0.0	2.6	2.2	0.7	6.3	1.9
Naviculales	4.6	0.5	0.0	0.9	13.3	2.0	11.1	4.8
Rhizosoleniales	1.6	0.0	0.0	0.0	3.9	0.9	9.2	4.6
Thalassionematales	2.3	3.5	0.0	2.6	8.4	22.9	0.0	18.7
Thalassiosirales	79.3	5.2	95.5	59.3	33.3	8.8	6.5	16.3
Triceratiales	1.4	0.0	0.0	0.3	6.4	0.5	0.0	0.0
Corethrales	0.0	0.0	0.0	0.0	0.0	3.3	0.0	0.8
Leptocylindrales	0.0	0.0	0.0	0.1	0.0	0.5	0.0	1.2
Other	2.3	0.0	2.6	0.0	2.0	0.0	1.3	0.4

*Note*: Low abundance species or zOTUs (<1% abundance) were grouped into “Other.”

**Table A4:** Summary of partial mantel tests showing Pearson's *R* correlations and *p* Values to explore the abiotic (environmental variables; Env) and biotic (Phytoplankton, Phyto) influence on bacterial community structure in the two size fractions for the top two surface depths (bacterial 16S rRNA and phytoplankton rbcL data set) or all depths (bacterial and plastid 16S rRNA).

Surface depths 20 m and 60–80 m		
Comparison: 16S rRNA	0.8–3 µm	>3 µm
Bacteria × Phyto | Env	0.4585*	0.4373*
Bacteria × Env | Phyto	−0.3868 ns	0.3581 ns
Phyto × Env | Bacteria	0.7273**	0.2257 ns
Comparison: rbcL	0.8–3 µm	>3 µm
Bacteria × Phyto | Env	0.6021**	0.4784*
Bacteria × Env | Phyto	−0.5317 ns	0.06923 ns
Phyto × Env | Bacteria	0.816**	0.6121**
**Depths 20 m, 60–80, 150, 300 m**		
Comparison: 16S rRNA	0.8–3 µm	>3 µm
Bacteria × Phyto | Env	0.4365**	0.487**
Bacteria × Env | Phyto	0.6116**	0.787**
Phyto × Env | Bacteria	0.2116*	−0.1679 ns

*Note*: Tests are presented as the 2 matrices being compared (e.g., Bacteria × Phyto) while controlling for the effects of the third matrix (e.g., |Env).

Abbreviation: ns, nonsignificant.

** *p* < 0.01

* *p* < 0.05.

**Table A5:** Proportional abundance of bacterial zOTUs (out of total bacterial zOTUs) correlating to phytoplankton zOTUs and proportional abundance of phytoplankton zOTUs correlating to bacterial zOTUs (out of total phytoplankton zOTUs) for the significantly correlated zOTUs in Figure 6.

Proportional abundance bacterial zOTUs correlating to phytoplankton zOTUs				Proportional abundance phytoplankton zOTUs correlating to bacterial zOTUs			
Sample	bact zOTUs (0.8–3 µm)	bact zOTUs (>3 µm)	Sum bact zOTUs	Sample	phyto zOTUs (0.8–3 µm)	phyto zOTUs (>3 µm)	Sum phyto zOTUs
A3.08.1	38.75	10.73	49.48	A3.08.1	70.87	12.73	83.60
A3.08.2	37.50	10.30	47.80	A3.08.2	63.26	18.60	81.86
E5.08.1	22.39	9.29	31.68	E5.08.1	35.59	2.74	38.33
E5.08.2	44.72	5.22	49.94	E5.08.2	58.94	4.70	63.65
F.08.1	39.11	6.97	46.08	F.08.1	78.34	3.18	81.52
F.08.2	43.28	3.84	47.13	F.08.2	88.01	1.35	89.36
R.08.1	38.42	5.66	44.08	R.08.1	87.93	1.99	89.92
R.08.2	32.62	6.57	39.19	R.08.2	89.45	0.26	89.72
A3.3.1	13.89	30.62	44.51	A3.3.1	9.65	77.49	87.14
A3.3.2	18.64	21.37	40.01	A3.3.2	7.67	78.88	86.54
E5.3.1	22.40	20.23	42.63	E5.3.1	2.97	5.87	8.84
E5.3.2	22.80	16.85	39.65	E5.3.2	5.10	17.81	22.91
F.3.1	19.34	11.63	30.97	F.3.1	14.52	45.31	59.83
F.3.2	20.21	16.28	36.49	F.3.2	23.88	42.27	66.15
R.3.1	23.62	19.51	43.12	R.3.1	37.29	46.38	83.68
R.3.2	11.35	35.30	46.65	R.3.2	28.27	52.64	80.91

## APPENDIX 3

Illumina data analysis

16S rRNA reads preprocessing and analysis using USEARCH v8/v9/11 unless QIIME v 1.9 or MOTHUR indicated.

*Sequence preprocessing*

1. Merge pairs (0 mismatches in overlap region)
  usearch8 ‐fastq_mergepairs 141003_SN1126_A_L001_HCI‐2_R1.fastq ‐reverse 141003_SN1126_A_L001_HCI‐2_R2.fastq ‐fastq_truncqual 3 ‐fastq_maxdiffs 0 ‐fastqout merged0.fastq
2. Quality filtering with usearch8 (maxee 1.0)
  usearch8 ‐fastq_filter merged0.fastq ‐fastq_maxee 1.0 ‐fastaout reads0_filt.fasta ‐fasta_cols 0
3. Separate eubacterial 16S reads in the forward sense using the 341F primer (ligation on PCR was not directional so reads appear in both orientations)
  grep ‐B1 "^.\{7\}CCTACGGG.GGC.GCAG" reads0_filt.fasta > eubfor0.fasta
4. Separate eubacterial 16S reads in the reverse sense using the 806R primer
  grep ‐B1 "GACTAC.GGGTATCTAATCC" reads0_filt.fasta > eubrev0.fasta
5. Reverse complement reverse reads with QIIME command
  adjust_seq_orientation.py ‐i eubrev0.fasta ‐r
6. Combine forward and reverse complemented reads in one file
  cat eubrev0_rc.fasta eubfor0.fasta > euball0.fasta
7. Remove the double hyphen that is introduced at the beginning of the sequence during the reverse complementing (otherwise the demultiplexing only assigns the forward reads)
  sed s'/\‐‐//g' euball0.fasta > eub0.fasta
8. Demultiplex reads using QIIME command to parse out reads to sample using the mapping file with no mismatches in barcode allowed
  demultiplex_fasta.py ‐f eub0.fasta ‐m mapping_file_eub.txt ‐b 7 ‐e 0.5 ‐‐retain_unassigned_reads
9. Parse out reads to the different projects: KEOPS2 and MOCK data set using the QIIME command
  extract_seqs_by_sample_id.py ‐i demultiplexed_seqs.fna ‐o keops2.fasta ‐m mapping_file_eub.txt ‐s "Project:KEOPS2"
  extract_seqs_by_sample_id.py ‐i demultiplexed_seqs.fna ‐o mock.fasta ‐m mapping_file_eub.txt ‐s "Project:MOCK"
10. Trim off primer sequences with MOTHUR allowing only exact matches to primers
  File: 16Soligos.txt
  forwardCCTACGGGNGGCWGCAG
  reverseGACTACHVGGGTATCTAATCC
  mothur > trim.seqs(fasta=keops2.fasta, oligos=16Soligos.txt)
  mothur > trim.seqs(fasta=mock.fasta, oligos=16Soligos.txt)
  *Identification of zOTUs (USEARCH‐32 v11)*
11. Dereplicate sequences
  usearch ‐fastx_uniques keops2.trim.fasta ‐fastaout keops2.uniques2.fasta ‐sizeout ‐relabel Uniq
12. Denoise using the UNOISE3 algorithm
  usearch ‐unoise3 keops2.uniques2.fasta ‐zotus keops2_zotus.fasta
13. Map reads to zOTUs (note that for correct mapping, the read sample names should not contain a period as a delimiter since this is the delimiter expected after the sample name and before the read count, eg A3_08_1.23 for A3, 0.8 µm fraction, depth 1, read count 23)
  usearch ‐otutab keops2.trim.fasta ‐zotus keops2_zotus.fasta ‐otutabout keops2zotutab2.txt ‐biomout keops2zotutab2.json ‐mapout keops2zotusmap2.txt ‐notmatched keops2unmappedzotus2.fa ‐dbmatched keops2zotus_with_sizes2.fa ‐sizeout
  *Classification of 16S rRNA zOTUs (USEARCH‐64 v9)*
14. Classify zOTU sequences using the 16S rRNA SILVA database v.123 and the SINTAX classifier
  usearch9 ‐sintax keops2_zotus.fasta ‐db /users/west/silva_16s_v123 ‐tabbedout keops2.zotus.silva.sintax ‐strand plus ‐sintax_cutoff 0.8
15. Convert Sintax taxonomic classification into GreenGenes format (using Excel) for later export into R package Phyloseq by keeping columns 1 (zOTU name) and 4 (taxonomy) (eg k:Bacteria,p:Cyanobacteria,c:Chloroplast replaced with k__Bacteria;p__Cyanobacteria;c__Chloroplast)
16. Remove zOTUs without classification at Kingdom level from sintax classification, from zOTU sequences and zOTU Table (2 zOTUs removed)
  Save as keops2_zotus.mod.fasta, keops2zotutab2mod.txt and keops2.zotus.silva.sintax.mod.txt
  *Alignment of zOTUs (Mothur)*
17. Align retained sequences to the mothur 16S rRNA SILVA SEED alignment (version 132)
  mothur> align.seqs(candidate=keops2_zotus.mod.fasta, template=core_set_aligned.fasta.imputed, processors=8)
18. Inspect the alignment manually with AliView v 1.18 to identify spurious length zOTU sequences (<300 bp) and remove these and the zOTUs in the flipaccnos (not aligned) file from the alignment
  Save as keops2_zotus.mod.align.mod.txt
  *Filtering of zOTUs from the zOTU table*
19. Make a BIOM table from the corrected zOTU table (step 16)
  biom convert ‐‐table‐type="OTU table" ‐i keops2zotutab2mod.txt ‐o keops2.zotus.biom ‐‐to‐json
20. Add taxonomy to the BIOM table
  biom add‐metadata ‐‐sc‐separated taxonomy ‐‐observation‐header OTUID,taxonomy ‐‐observation‐metadata‐fp keops2.zotus.silva.sintax.mod.txt ‐i keops2.zotus.biom ‐o keops2.zotus.tax.biom
21. Remove the 17 spurious zOTU sequences (in the flip.accnos file or short length) identified in steps 17 and 18 (zOTUs names saved as list in file bad_Zotus.txt). (QIIME)
  filter_otus_from_otu_table.py ‐i keops2.zotus.tax.biom ‐o keops2.zotus.tax.filt.biom ‐e bad_Zotus.txt
22. Remove low count samples
  filter_samples_from_otu_table.py ‐i keops2.zotus.tax.filt.biom ‐o keops2.zotus.tax.filt2.biom ‐n 100
  *Prepare mapping file and tree for phyloseq object creation*
23. Sort sample order according to the mapping file
  sort_otu_table.py ‐i keops2.zotus.tax.filt2.biom ‐o keops2.zotus.tax.filt2.sort.biom ‐m mapping_file_keopsII_mod2.txt ‐s SampleOrder
24. Make a tree of zOTU sequences
  make_phylogeny.py ‐i keops2_zotus.mod.align.mod.txt ‐o keops2.zotus.repset.tre
25. Convert biom table to json format for input into R package phyloseq
  biom convert ‐i keops2.zotus.tax.filt2.sort.biom ‐o keops2zotus.json.biom ‐‐table‐type="OTU table" ‐‐to‐json
  *Create phyloseq object*
26. Import into phyloseq in R Studio (phyloseq and ape packages)

library(ape)

library(phyloseq)

keops2zotu_otutab <‐ import_biom("keops2zotus.json.biom", parseFunction=parse_taxonomy_greengenes, parallel=TRUE)

keops2map <‐ import_qiime_sample_data("mapping_file_keopsII_mod2.txt")

keops2zotutree <‐ read.tree("keops2.zotus.repset.tre")

keops2zotu <‐ merge_phyloseq(keops2zotu_otutab, keops2map, keops2zotutree)

Classification of Plastid sequences with PhytoRef

To obtain a more informative classification of the 16S rRNA chloroplast sequences than offered by the SILVA database v.123 database, we used the PhytoRef database (Decelle et al., 2015).

1. To be able to use this database with the sintax classifier, the headings of the PhytoRef taxonomy can be modified with the following unix script:
  cat PhytoRef_with_taxonomy.fasta | awk ‐F "|" '{if($1~/>/) {printf("%s;",$1); printf("tax=k:%s,",$2); printf("p:%s,",$4); printf("c:%s,",$5); printf("o:%s,",$7); printf("f:%s,",$9); printf("g:%s,",$10); printf("s:%s;",$11); printf("%s","\n")} else {print $0}}' > phytoref.sintax.txt
2. The 16S rRNA zOTUs identified as chloroplast sequences (step 14) are subsetted out to a new phyloseq object and the zOTU ID names are saved as text (phyloseq)
  keops2zotuchloro <‐ subset_taxa(keops2zotu, Class=="Chloroplast")
  listchlorozotus <‐ taxa_names(keops2zotuchloro)
3. The chloroplast zOTU sequences are extracted from the 16S rRNA fasta file using the zOTU IDs (QIIME)
  filter_fasta.py ‐f keops2_zotus.mod.fasta ‐o keops2zotuchloro.fasta ‐s listchlorozotus.txt
4. Classification with sintax
  usearch9 ‐sintax keops2zotuchloro.fasta ‐db phytoref.sintax.txt ‐tabbedout keops2zotuchloro.phytoref.sintax ‐strand plus ‐sintax_cutoff 0.8
5. Convert sintax taxonomic headers as in step 15 for later importation into phyloseq.
6. Export zOTU table from phyloseq
  write.csv(otu_table(keops2zotuchloro), file="keops2zotuchlorootutab.csv")
  check the table and save it as txt
7. Make biom file from zOTU table, add taxonomy and convert to json format for phyloseq reimportation
  biom convert ‐‐table‐type="OTU table" ‐i keops2zotuchlorootutab.txt ‐o keops2zotuchloro.biom ‐‐to‐json
  biom add‐metadata ‐‐sc‐separated taxonomy ‐‐observation‐header OTUID,taxonomy ‐‐observation‐metadata‐fp keops2zotuchloro.phytoref.sintax.mod.txt ‐i keops2zotuchloro.biom ‐o keops2zotuchlorotax.biom
  biom convert ‐i keops2zotuchlorotax.biom ‐o keops2zotuchlorotaxjson.biom ‐‐table‐type="OTU table" ‐‐to‐json
8. Reimport into phyloseq

keops2zotuchlorootutab <‐ import_biom("keops2zotuchlorotaxjson.biom", parseFunction=parse_taxonomy_greengenes, parallel=TRUE)

keops2zotuchloro <‐ merge_phyloseq(keops2zotuchlorootutab, keops2map, keops2zotutree)

Sequence preprocessing of *rbc*L sequence reads (using USEARCH v8/v9 unless QIIME v 1.9 or MOTHUR indicated)

1. rbcL sequences were essentially processed as the 16S rRNA reads from step 3 using the rbcL primers until step 8 with primer removal achieved with the custom python script strip_primers_exclude.py from Tony Walterst (https://gist.github.com/walterst).
2. zOTU identification was performed the same as for the 16S rRNA reads from steps 11‐13.
3. zOTU sequences were aligned and inspected with AliView and 5 short sequences were removed.
4. zOTUs were classified using blastn with further manual taxonomic refinement using the WoRMS database (WoRMS Editorial Board, 2016).
